# Acute kidney disease after acute kidney injury: incidence, risk factors, and association with *de novo* chronic kidney disease

**DOI:** 10.1080/0886022X.2026.2745291

**Published:** 2026-10-15

**Authors:** Sujia Hu, Xingyang Zhao, Yichun Cheng, Shuwang Ge

**Affiliations:** Department of Nephrology, Tongji Hospital, Tongji Medical College, Huazhong University of Science and Technology, Wuhan, Hubei, China

**Keywords:** Acute kidney injury, acute kidney disease, chronic kidney disease, incidence, risk factors

## Abstract

Acute Kidney Disease (AKD) is defined as acute kidney injury (AKI) persisting for 7 to 90 days and represents a window for interventions to improve renal outcomes. However, data on AKD remain underexplored. This retrospective cohort study at Tongji Hospital, China, included 23,868 adult patients who survived at least one AKI episode between January 2015 and April 2024. Using electronic medical record data, we aimed to quantify the incidence of AKD among AKI survivors, identify independent risk factors, and evaluate the association between AKD and *de novo* chronic kidney disease (CKD). Among the cohort, 10,884 patients (45.6%) progressed to AKD. Multivariable logistic regression revealed that advanced age, preexisting hypertension, heart failure, cirrhosis, recent diuretic use, hepatorenal syndrome, higher baseline estimated glomerular filtration rate, higher AKI stage, and dialysis-requiring AKI were independent risk factors for AKD. Fine-Gray competing-risk analysis accounting for acute-phase mortality confirmed that higher AKI severity was independently associated with AKD progression. In a subcohort of 6597 patients without preexisting CKD who had at least one year of follow-up, 19.8% developed *de novo* CKD. Cox proportional hazards analysis demonstrated that patients with AKD had a significantly increased risk of *de novo* CKD compared to those without AKD (HR 1.92, 95% CI 1.70–2.17). These findings highlight the high proportion of AKD among patients with AKI, and emphasize the importance of early detection and targeted management for high-risk groups. It is critical to strengthen post-discharge renal function monitoring and intervention strategies for AKI patients to improve long-term renal outcomes.

## Introduction

Acute kidney injury (AKI) refers to a sharp decline in renal function within 7 days, usually leading to increased in-hospital mortality and prolonged hospitalization, increasing the economic burden on the healthcare system [1,2], and is associated with a poor prognosis of cardiovascular events [3], and an increased risk of death [4]. In recent years, the incidence of AKI has been increasing worldwide [5,6], affecting 7%–18% of hospitalized patients and 30%–70% of critically ill patients [7]. There are varying degrees of renal recovery after AKI, and those who have not fully recovered by three months may progress to chronic kidney disease (CKD) [8]. A prospective study reported that, within three years after AKI, 24.6% of patients developed CKD [9].

However, a subset of patients develop abnormalities in renal function or morphology that fall short of the conventional definitions for CKD or AKI but still warrant clinical management. To promote standardized management of this group of patients, the Kidney Disease: Improving Global Outcomes (KDIGO) introduced the concept of Acute Kidney Disease (AKD) in 2012 [10]. Subsequently, the Acute Disease Quality Initiative (ADQI) 16 Workgroup further refined the staging of AKD [11]. According to the KDIGO framework, AKD broadly refers to abnormalities of kidney function and/or structure lasting for up to 3 months and encompasses AKI as well as subacute kidney abnormalities that do not meet the timing or severity criteria for AKI [12]. In the post-AKI setting, the ADQI 16 Workgroup further operationalized AKD as kidney dysfunction persisting for 7–90 days after the index AKI episode, including persistence of at least KDIGO AKI stage 1 severity during that interval [11]. Nevertheless, the framework also encompasses ‘Stage 0’ to monitor patients with clinical resolution who still carry long-term prognostic risks [11]. This interval represents a critical period for renal repair and is considered a window of time in which interventions can be implemented to alter AKI outcomes [13]. In the post-AKI context, current studies often describe AKI-AKD-CKD as a continuum to monitor the clinical progression of renal function following AKI [14–16].

Recent studies have substantially advanced the epidemiological understanding of AKD. Systematic reviews and meta-analyses have shown that AKD is common and is associated with increased risks of mortality, *de novo* CKD, and kidney failure [17]. However, reported incidence estimates vary considerably across studies, partly because of differences in AKD definitions, ascertainment strategies, study populations, and whether AKD is evaluated with or without preceding AKI. In particular, less is known about the clinical profile of patients who develop AKD following AKI in large Chinese clinical cohorts. Addressing these gaps is crucial for optimizing post-AKI surveillance, identifying high-risk patients, and guiding targeted management strategies.

We conducted a retrospective study in China to explore the epidemiological characteristics of AKD in patients following AKI. Our objectives were to examine the incidence of AKD and CKD after AKI, identify independent predictors for AKD progression in AKI patients, and assess the relationship between AKD following AKI and *de novo* CKD. We hypothesized that higher AKI severity increases AKD risk, and subsequent incomplete AKD recovery further drives progression to *de novo* CKD.

## Methods

### Study design and participants

We conducted a retrospective cohort analysis using data extracted from the Tongji Biobank at Tongji Hospital, Tongji Medical College, Huazhong University of Science and Technology (Wuhan, China). This large-scale single-center biomedical repository has accumulated multi-dimensional clinical and experimental data since 1980 [18], and all data access and usage complied with its standardized ethical review and data management protocols. Demographics, diagnoses, medication records, laboratory results, ICU admissions, surgical procedures, and in-hospital death were retrieved from the hospital electronic medical record system.

Patients diagnosed with AKI during inpatient hospitalization, outpatient visits, or ambulatory ward visits at Tongji Hospital, Wuhan, between 1 January 2015, and 30 April 2024, were included. For individuals with more than one AKI event within the study window, the first occurrence was defined as the index episode. For the analysis of AKD incidence and predictors, the exclusion criteria were age <18 years (*N* = 4097), confirmed end-stage renal disease (*N* = 8940, CKD stage 5/uremia or a history of dialysis or kidney transplantation), and absence of serum creatinine data (*N* = 14,172) required for AKD identification. After exclusions, 26,253 patients were included in the analysis, comprising 23,868 who survived beyond 7 days after AKI onset and 2385 who died within 7 days. The primary analysis was restricted to survivors beyond 7 days, whereas data from those who died within 7 days were included only in sensitivity analyses. Furthermore, for the analysis of the association between AKD and *de novo* CKD, patients with a prior CKD diagnosis (*N* = 5679) and those lacking serum creatinine data necessary for *de novo* CKD identification (*N* = 11,592) were further excluded. To maximize statistical power, all eligible patients within the study period were included. It is worth noting that many patients failed to undergo regular creatinine monitoring or completed their follow-up at local primary care facilities. Consequently, their follow-up serum creatinine (SCr) data were not captured by our electronic medical records, resulting in their exclusion. The participant selection process is illustrated in Figure 1.

**Figure 1.**
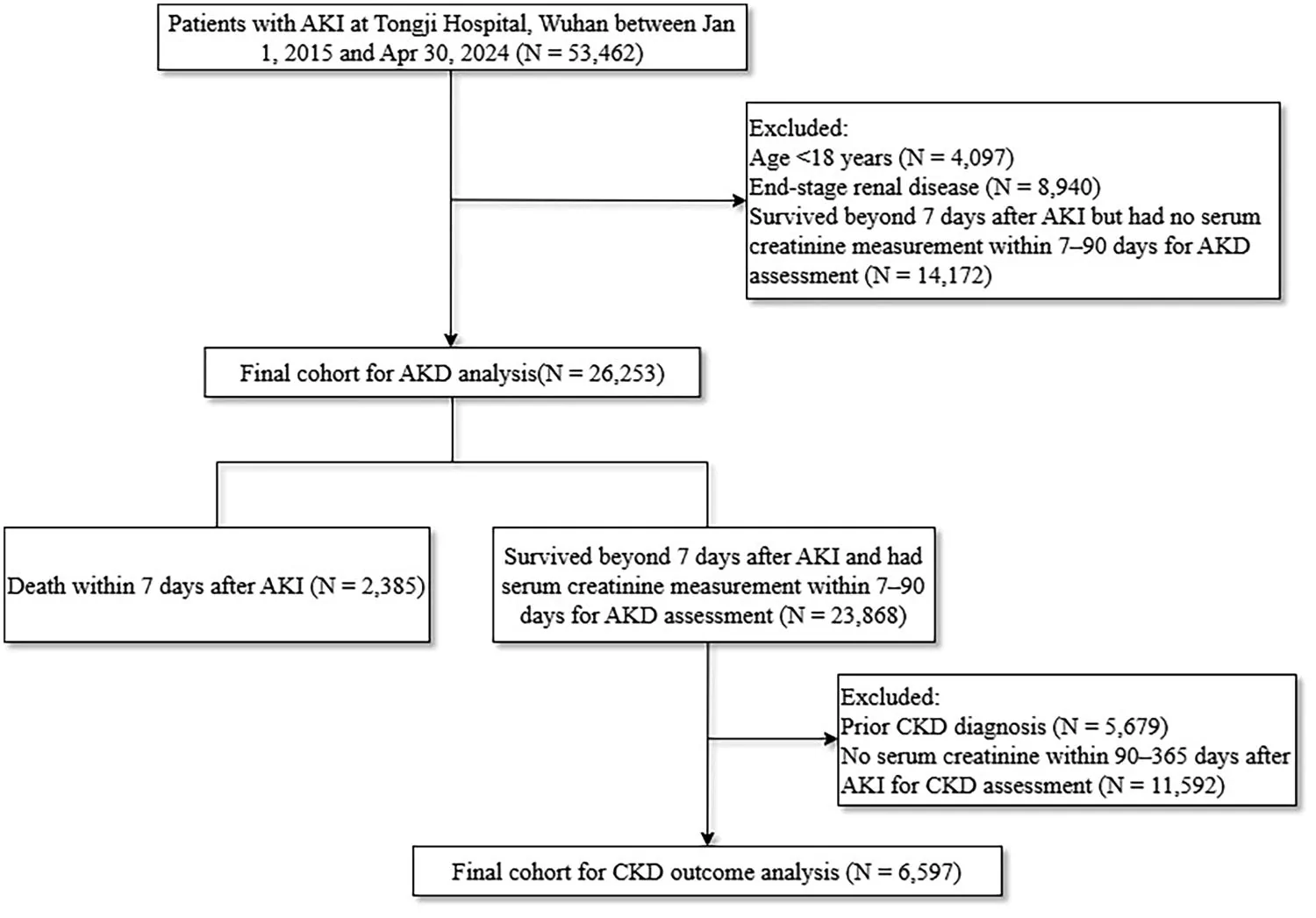
Flowchart of participants selection.

In accordance with the Declaration of Helsinki, the study protocol was reviewed and approved by the Ethics Committee of Tongji Hospital, Tongji Medical College, Huazhong University of Science and Technology (Project No. TJ-IRB202511019). The requirement for informed consent was waived by the committee, as this was a retrospective study and all personal information was anonymized.

### Definitions

Diagnosis and staging of AKI followed KDIGO guidance [10]. AKI was considered present when SCr increased by ≥26.5 μmol/L within 48 h or reached ≥1.5× the baseline within 7 days. Staging used the highest SCr recorded during the first 7 days after onset: stage 1 (1.5–1.9× baseline or ≥26.5 μmol/L rise), stage 2 (2.0–2.9×), and stage 3 (≥3.0×). We used an algorithm based on dynamic SCr to determine the occurrence of AKI [19]. First, all SCr measurements for each patient were sorted in ascending order by collection time. At any given time point *t*, the dynamic baseline was defined as the average SCr level within the 7 days prior to *t*. Each available SCr measurement within the 7 days following *t* was then compared with this baseline. The earliest date on which the SCr change met the KDIGO criteria was identified as the AKI episode date. When multiple SCr tests were performed on the same day, the maximum value was used. Urine-output data were unavailable; therefore, creatinine-based criteria alone were applied. AKD was defined as persistence of AKI stage ≥1 for 7–90 days after the index AKI episode, which required at least one documented serum creatinine measurement during this interval. AKD staging mirrored the AKI SCr multiple thresholds and was applied to the highest SCr recorded 7 to <90 days after AKI onset relative to the same baseline SCr [11]. To align with the ADQI consensus framework, the AKD spectrum was categorized into stages 3 (≥3.0-fold baseline), 2 (2.0- to 2.9-fold baseline), 1 (1.5- to 1.9-fold baseline), and 0 C (1.0- to 1.4-fold baseline), while individuals with a follow-up SCr ratio < 1.0 relative to baseline were categorized as stage 0 A/0B. CKD was defined according to the KDIGO guidelines [12]. Because of data constraints, CKD required evidence of kidney abnormality for ≥3 months, operationalized as eGFR <60 mL/min/1.73 m^2^ or persistent kidney-damage markers: dipstick protein ≥1+, urine albumin-to-creatinine ratio (UACR) ≥30 mg/g, or 24-h urinary protein excretion >0.15 g. eGFR was calculated using the 2021 Chronic Kidney Disease Epidemiology Collaboration (CKD-EPI) creatinine equation. Prior CKD was operationalized as any documented fulfillment of the aforementioned criteria before the index AKI date. *De novo* CKD was defined as meeting the above criteria at least three months after the occurrence of AKI.

### Variables

We extracted demographics (age, sex), comorbidities, precipitants and etiologies, medication exposure, and laboratory indices from the electronic medical record. Comorbidities considered were hypertension, diabetes mellitus, cancer, coronary artery disease, congestive heart failure, cirrhosis, chronic obstructive pulmonary disease (COPD). Precipitants and etiologies refer to sepsis, hypovolemia, obstruction, nephrotoxins, cardiorenal syndrome, or hepatorenal syndrome. Exposure to nephrotoxins was defined as the prescription of any potentially nephrotoxic medications within 7 days prior to the index AKI onset, according to a published literature review [20]. Drug screening included renin-angiotensin-aldosterone system inhibitors, including Angiotensin-Converting Enzyme Inhibitor (ACEI) and Angiotensin II Receptor Blocker (ARB), diuretics, metformin, lipid-lowering statins, and antibiotics. Medication was defined as any prescription within 7 days prior to the AKI episode. Laboratory measurements included serum creatinine (SCr) and estimated glomerular filtration rate (eGFR). eGFR was analyzed both as a continuous variable and as a categorical variable, grouped as <30, 30–44, 45–59, 60–89, and ≥90 mL/min/1.73 m^2^. Additionally, based on previous literature, we also included ICU admission, surgery, dialysis-requiring AKI (defined as the initiation of acute dialysis support *via* temporary vascular access during the index AKI episode in individuals with no prior history of end-stage renal disease (ESRD)), hospital length of stay (for patients who were not hospitalized, the value was coded as 0), AKI stage as candidate variables.

### Statistical analysis

Baseline tables were utilized to comprehensively present the characteristics of patients across different clinical groups. To describe the study cohort, continuous variables were summarized as median [interquartile range (IQR)] and compared between groups using the Wilcoxon rank-sum test for two groups or the Kruskal–Wallis test for three or more groups. Categorical variables were presented as counts (percentages) and examined using the chi-square test. Given the large sample size of the present study, which can render *p* value overly sensitive to clinically negligible differences, the standardized mean difference (SMD) was additionally calculated alongside P-value to rigorously evaluate the magnitude of group imbalances. An SMD value greater than 0.10 was predefined as the threshold indicating meaningful baseline disparity. Regarding data completeness, baseline continuous variables (including age, baseline eGFR, and hospital length of stay) and all binary clinical covariates were entirely populated with zero missingness within the electronic medical record system. Furthermore, to address potential selection bias introduced by historical follow-up availability, a comprehensive comparative analysis of baseline characteristics was performed separately within both the AKD analytical cohort and the CKD outcome cohort between the enrolled individuals and those excluded due to unavailable creatinine records.

Risk factors for transition from AKI to AKD were explored with logistic regression. Candidate covariates were first screened in univariable models and those with *p* < 0.1 were entered into the multivariable model. Considering that early mortality may mask the true incidence risk of AKD, leading to an underestimation of its risk factors, sensitivity analyses were conducted by employing the Fine-Gray model to evaluate the association between AKI stages and the development of AKD, treating death within 7 days after AKI onset as a competing event. Additionally, the variance inflation factors (VIFs) were inspected to rule out problematic multicollinearity.

For the *de novo* CKD outcome analysis, the longitudinal follow-up commenced at day 90 post-AKI. To evaluate long-term kidney outcomes, cumulative incidence curves were plotted to compare the risk of *de novo* CKD among patients with varying stages of AKD. The association of AKD and its stages with *de novo* CKD was quantified using Cox proportional hazards models. Variables meeting *p* < 0.1 in univariate analyses were retained in multivariable Cox models. For stratified analyses, patients were categorized by pre-AKI surgery, major comorbidities, AKI cardiorenal syndrome, baseline eGFR, AKI stage, and dialysis-requiring AKI.

All computations were carried out in R (version 4.4.2; R Foundation, Vienna). A two-sided *p* < 0.05 denoted statistical significance.

### Reporting guideline

This cohort study was reported in accordance with the STROBE reporting guidelines, and the completed STROBE checklist is provided in the Supplementary Materials.

## Results

### Description of the cohort

As shown in Table 1, of 26,253 patients with AKI, 23,868 survived beyond day 7. Overall, 2385 of 26,253 patients (9.1%) died within 7 days after AKI onset. A comparison of baseline characteristics between included patients and those excluded due to missing serum creatinine measurements is detailed in Table S1. Although SMDs across most baseline comorbidities were small, patients in the included cohort had substantially longer hospital stays and higher rates of surgery and diuretic exposure. Of these survivors, 45.6% (10,884/23,868) developed AKD (ADQI stages 1–3), including 40.6% at stage 1 (4420/10,884), 27.6% at stage 2 (3000/10,884), and 31.8% at stage 3 (3464/10,884). Among patients without AKD (ADQI stage 0, *n* = 12,984), 34.9% (4528/12,984) were classified as stage 0 A/0B and 65.1% (8456/12,984) as stage 0 C. An additional 2,385 patients died within 7 days of the AKI episode; these early deaths occurred in older patients (median age 64 [52–75] vs. 57 [47–66] years for survivors) and more frequently involved ICU admission (23.6%) or sepsis (18.4%).

**Table 1.** Baseline characteristics of participants enrolled in the cohort.

Characteristics	AKD Stage 1–3	AKD Stage 0	*p* value	SMD	Death within 7 days after AKI
N	10884	12984			2385
Age (median [IQR])	56.0 [46.0, 65.0]	57.0 [48.0, 66.0]	<0.001	0.069	64.0 [52.0, 75.0]
Sex, male (%)	6884 (63.2)	8641 (66.6)	<0.001	0.069	1657 (69.5)
ICU admission (%)	671 (6.2)	1250 (9.6)	<0.001	0.129	562 (23.6)
Surgery (%)	5841 (53.7)	6701 (51.6)	0.002	0.041	494 (20.7)
Hypertension (%)	3637 (33.4)	4538 (35.0)	0.013	0.032	857 (35.9)
Diabetes (%)	1636 (15.0)	2117 (16.3)	0.007	0.035	491 (20.6)
Cancer (%)	3917 (36.0)	4827 (37.2)	0.06	0.025	892 (37.4)
Coronary artery disease (%)	2338 (21.5)	2534 (19.5)	<0.001	0.049	482 (20.2)
Congestive heart failure (%)	2490 (22.9)	2202 (17.0)	<0.001	0.149	707 (29.6)
Cirrhosis (%)	977 (9.0)	995 (7.7)	<0.001	0.048	194 (8.1)
COPD (%)	262 (2.4)	304 (2.3)	0.772	0.004	121 (5.1)
Sepsis (%)	704 (6.5)	900 (6.9)	0.162	0.019	440 (18.4)
Hypovolemia (%)	174 (1.6)	250 (1.9)	0.064	0.025	149 (6.2)
Obstruction (%)	128 (1.2)	235 (1.8)	<0.001	0.052	21 (0.9)
Cardiorenal syndrome (%)	451 (4.1)	605 (4.7)	0.058	0.025	194 (8.1)
Hepatorenal syndrome (%)	333 (3.1)	208 (1.6)	<0.001	0.097	182 (7.6)
Nephrotoxins (%)	7518 (69.1)	8555 (65.9)	<0.001	0.068	1464 (61.4)
ACEI/ARB (%)	1260 (11.6)	1482 (11.4)	0.71	0.005	173 (7.3)
Diuretic (%)	7237 (66.5)	7803 (60.1)	<0.001	0.133	1498 (62.8)
Metformin (%)	175 (1.6)	223 (1.7)	0.543	0.009	40 (1.7)
Statin (%)	1639 (15.1)	1952 (15.0)	0.972	0.001	311 (13.0)
Antibiotics (%)	9011 (82.8)	10862 (83.7)	0.077	0.023	2130 (89.3)
Baseline eGFR, mL/min/1.73 m^2^ (median [IQR])	108.4 [83.0, 125.5]	93.7 [60.3, 108.8]	<0.001	0.53	91.8 [61.2, 107.0]
Baseline eGFR group, mL/min/1.73 m^2^ (%)			<0.001	0.364	
<30	527 (4.8)	1193 (9.2)			167 (7.0)
30–44	547 (5.0)	953 (7.3)			183 (7.7)
45–59	587 (5.4)	1079 (8.3)			228 (9.6)
60–89	1474 (13.5)	2743 (21.1)			563 (23.6)
≥90	7749 (71.2)	7016 (54.0)			1244 (52.2)
Peak AKI stage within 7 days post AKI (%)			<0.001	0.97	
Stage 1	4869 (44.7)	10962 (84.4)			1204 (50.5)
Stage 2	2886 (26.5)	1633 (12.6)			631 (26.5)
Stage 3	3129 (28.7)	389 (3.0)			550 (23.1)
Dialysis-requiring AKI (%)	460 (4.2)	146 (1.1)	<0.001	0.193	37 (1.6)
Hospital length of stay (median [IQR])	23.0 [15.0, 33.0]	20.0 [13.0, 30.0]	<0.001	0.162	8.0 [4.0, 15.0]
AKD stage (%)			–	–	–
Stage 0 A/0B	–	4528 (34.9)			
Stage 0 C	–	8456 (65.1)			
Stage 1	4420 (40.6)	–			
Stage 2	3000 (27.6)	–			
Stage 3	3464 (31.8)	–			

Data are presented as median [interquartile range (IQR)] for continuous variables or number (percentage) for categorical variables.

SMD: Standardized Mean Difference; ICU: Intensive Care Unit; ACEI: Angiotensin-converting enzyme inhibitors; ARB: Angiotensin II receptor blockers; COPD: Chronic Obstructive Pulmonary Disease; eGFR: Estimated glomerular filtration rate; SCr: Serum creatinine.

While demographic characteristics such as age and sex were largely comparable between the two groups (SMD < 0.1), patients in stages 1–3 exhibited substantially higher baseline eGFR (108.4 [83.0–125.5] vs. 93.7 [60.3–108.8] mL/min/1.73 m^2^), and experienced longer hospital stays (23 [15–33] vs. 20 [13–30] days). Regarding clinical etiologies and disease severity, differences in most underlying acute insults remained small in effect size (SMD < 0.1); however, compared with patients in stage 0, those in stages 1–3 had a meaningfully higher prevalence of pre-AKI diuretic use (66.5% vs. 60.1%, SMD = 0.133) and dialysis-requiring AKI (4.2% vs. 1.1%, SMD = 0.193). Furthermore, these patients were more likely to experience higher AKI stages within the first 7 days. Baseline characteristics stratified by AKD stage are shown in Table S2.

### Risk factors associated with AKD progression

The risk factors associated with progression to AKD among the tested individuals are summarized in Table 2. In the multivariable logistic regression model, progression to AKD occurred more often in older individuals and in those with hypertension, congestive heart failure, or cirrhosis. Among acute precipitants and clinical etiologies, hepatorenal syndrome was independently associated with an increased likelihood of AKD progression (OR 1.95, 95% CI 1.59–2.39). Medication exposure also played an important role, with the use of diuretics within 7 days prior to the AKI episode significantly associated with an increased likelihood (OR 1.14, 95% CI 1.07–1.21). Higher baseline eGFR (OR 1.02, 95% CI 1.01–1.02), higher AKI stage (OR 3.72 for stage 2; OR 13.16 for stage 3), and dialysis-requiring AKI (OR 3.35, 95% CI 2.67–4.21) were also independently associated with progression to AKD. By contrast, male sex, ICU admission, surgical procedures, and exposure to antibiotics were linked to a reduced likelihood of AKD development.

**Table 2.** Univariable and multivariable logistic regression of risk factors for AKD progression in AKI patients, excluding deaths within 7 days.

Risk factors	Crude model^a^		Adjusted model^b^	
	OR (95% CI)	*p* value	OR (95% CI)	*p* value
Age	1.00 (0.99–1.00)	<.001	1.01 (1.00–1.01)	<.001
Sex, male	0.86 (0.82–0.91)	<.001	0.91 (0.85–0.97)	0.002
ICU admission	0.62 (0.56–0.68)	<.001	0.77 (0.69–0.87)	<.001
Surgery	1.09 (1.03–1.14)	0.002	0.71 (0.66–0.75)	<.001
Hypertension	0.93 (0.89–0.99)	0.013	1.13 (1.06–1.22)	<.001
Diabetes	0.91 (0.85–0.97)	0.007	1.09 (1.00–1.18)	0.05
Cancer	0.95 (0.90–1.00)	0.058	1.06 (0.99–1.13)	0.083
Coronary artery disease	1.13 (1.06–1.20)	<.001	1.04 (0.96–1.14)	0.33
Congestive heart failure	1.45 (1.36–1.55)	<.001	1.46 (1.34–1.59)	<.001
Cirrhosis	1.19 (1.08–1.30)	<.001	1.21 (1.08–1.35)	<.001
COPD	1.03 (0.87–1.22)	0.739		
Sepsis	0.93 (0.84–1.03)	0.154		
Hypovolemia	0.83 (0.68–1.01)	0.057	0.97 (0.77–1.21)	0.769
Obstruction	0.65 (0.52–0.80)	<.001	0.83 (0.64–1.06)	0.139
Cardiorenal syndrome	0.88 (0.78–1.00)	0.054	1.05 (0.90–1.22)	0.531
Hepatorenal syndrome	1.94 (1.63–2.31)	<.001	1.95 (1.59–2.39)	<.001
Nephrotoxins	1.16 (1.09–1.22)	<.001	0.97 (0.91–1.03)	0.316
ACEI/ARB	1.02 (0.94–1.10)	0.695		
Diuretic	1.32 (1.25–1.39)	<.001	1.14 (1.07–1.21)	<.001
Metformin	0.94 (0.77–1.14)	0.51		
Statin	1.00 (0.93–1.08)	0.957		
Antibiotics	0.94 (0.88–1.01)	0.074	0.83 (0.76–0.90)	<.001
Baseline eGFR, mL/min/1.73 m^2^	1.02 (1.01–1.02)	<.001	1.02 (1.01–1.02)	<.001
**Baseline eGFR group, mL/min/1.73 m^2^ (versus <30)**				
30–44	1.30 (1.12–1.50)	<.001	1.30 (1.12–1.50)	<.001
45–59	1.23 (1.07–1.42)	0.004	1.23 (1.07–1.42)	0.004
60–89	1.22 (1.08–1.37)	0.001	1.22 (1.08–1.37)	0.001
≥90	2.50 (2.25–2.78)	<.001	2.50 (2.25–2.78)	<.001
**Peak AKI stage within 7 days post AKI(versus Stage 1)**				
Stage 2	3.98 (3.71–4.27)	<.001	3.72 (3.46–4.00)	<.001
Stage 3	18.11 (16.21–20.23)	<.001	13.16 (11.73–14.77)	<.001
Dialysis-requiring AKI	3.88 (3.22–4.68)	<.001	3.35 (2.67–4.21)	<.001

ICU: Intensive Care Unit; ACEI: Angiotensin-converting enzyme inhibitors; ARB: Angiotensin II receptor blockers; COPD: Chronic Obstructive Pulmonary Disease; eGFR: Estimated glomerular filtration rate; SCr: Serum creatinine.

^a^The Crude model is unadjusted.

^b^Variables with a *p* value <0.1 in univariate analyses were included in multivariate logistic regression models to adjust for potential confounders.

To account for the competing risk of mortality during the acute phase, a Fine-Gray competing-risk regression analysis was conducted (Table S3). This model confirmed that higher AKI severity remained strongly and independently associated with subsequent AKD progression (sHR 2.33 for stage 2 and 3.54 for stage 3, both *p* < 0.001).

### Association of AKD with 1-year de novo CKD

A comparison between included and excluded patients due to missing follow-up creatinine for CKD assessment is presented in Table S4. Notably, the included cohort was younger and enriched for patients with underlying cancer, whereas excluded patients had higher baseline rates of ICU admission, congestive heart failure, and sepsis. Characteristics by occurrence of *de novo* CKD are summarized in Table S5. Among 6597 patients in the cohort, 1308 (19.8%) developed *de novo* CKD during follow-up. As shown in Figure 2, the cumulative risk was consistently higher among those with AKD than among those without AKD. Furthermore, a clear stage-dependent increase in the risk of *de novo* CKD was observed with increasing AKD stage.

**Figure 2.**
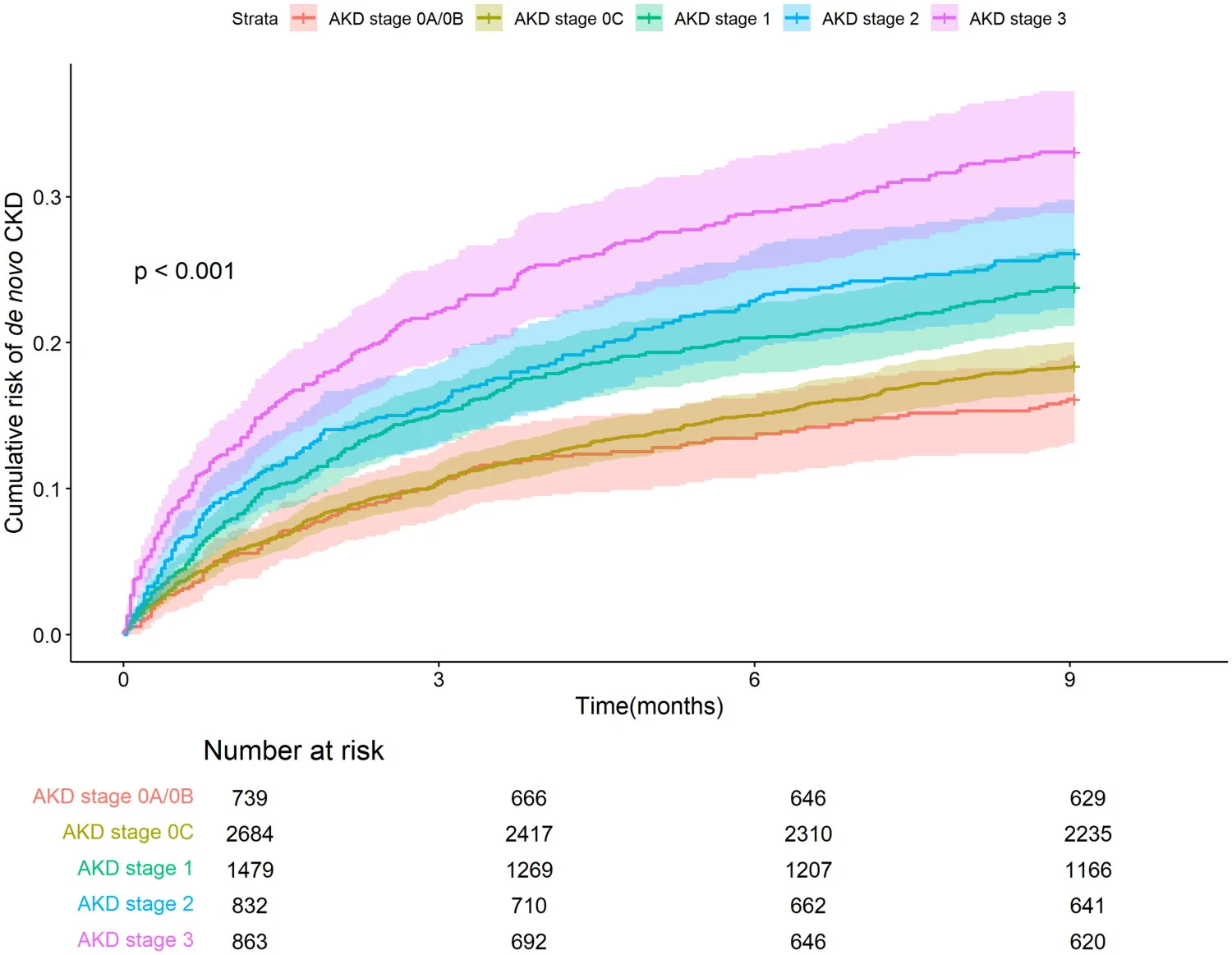
Cumulative risk of *de novo* CKD in AKI patients, stratified by AKD stage.

In univariable Cox models (Table S6), AKD predicted a higher 1-year risk of *de novo* CKD (HR 1.51; 95% CI 1.36–1.69; *p* < 0.001). As shown in Table 3, after multivariable adjustment for variables with *p* < 0.10, AKD remained independently associated with *de novo* CKD (HR 1.92; 95% CI 1.70–2.17; *p* < 0.001). The risk increased progressively across AKD stages (ranging from HR 1.74 for stage 0 C to HR 4.57 for stage 3).

**Table 3.** Multivariable Cox regression of AKD and AKD stages in relation to *de novo* CKD.

Characteristics	Crude model^a^			Adjusted model^b^		
	HR	95%CI	*p* value	HR	95%CI	*p* value
AKD	1.51	1.36-1.69	<0.001	1.92	1.70–2.17	<0.001
AKD Stages (versus Stage 0 A/0B)						
Stage 0 C	1.14	0.92–1.40	0.23	1.74	1.41–2.15	<0.001
Stage 1	1.48	1.19–1.83	<0.001	2.66	2.12–3.33	<0.001
Stage 2	1.62	1.28–2.05	<0.001	2.93	2.29–3.74	<0.001
Stage 3	2.08	1.66–2.61	<0.001	4.57	3.51–5.94	<0.001

^a^The Crude model is unadjusted.

^b^The Adjusted model controls for Age, Surgery, Hypertension, Diabetes, Coronary artery disease, Congestive heart failure, COPD, Sepsis, Cardiorenal syndrome, ACEI/ARB, Diuretic, Metformin, Statin, Baseline eGFR group, Peak AKI stage within 7 days post AKI, Dialysis-requiring AKI.

Subgroup and interaction analyses (Figure 3) indicated broadly consistent associations across patient strata. Pre-AKI surgery strengthened the association between AKD and *de novo* CKD (HR 2.52, 95% CI 2.12–2.99; interaction *p* = 0.001). The effect was also modified by sepsis (interaction *p* = 0.007), with a higher risk in patients without sepsis before AKI (HR 2.03, 95% CI 1.79–2.30). AKI stage also influenced this relationship, with the strongest association observed in patients with stage 2 AKI (HR 2.36, 95% CI 1.78–3.13). For other subgroups, no significant interactions were found, suggesting that the effect of AKD on CKD risk was largely consistent across these categories.

**Figure 3.**
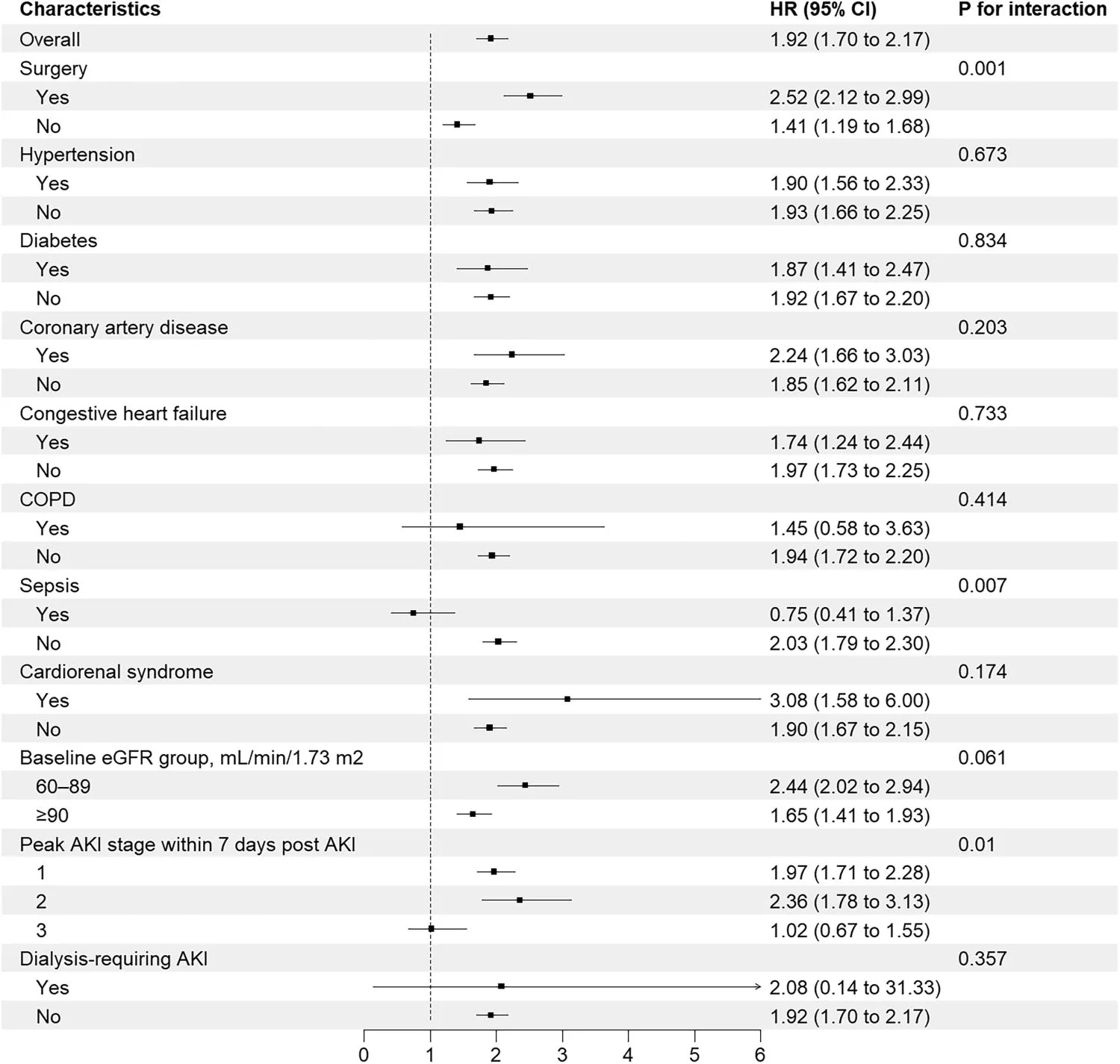
Subgroup analysis of the association between AKD and *de novo* CKD using Cox regression. Data are presented as the Hazard Ratio (95% confidence interval). HRs compare patients with AKD versus those without AKD within each subgroup. The models were adjusted for all covariates but not for the subgroup-specific variables.

## Discussion

In this retrospective study of 23,868 patients with AKI in China, 45.6% progressed to AKD stages 1–3 rather than recovering by 7 days after AKI onset, with a relatively balanced distribution across the three stages of AKD. Notably, among patients who did not meet the criteria for AKD stages 1–3, 65.1% were classified as ADQI stage 0 C, indicating subclinical non-recovery. Univariate and multivariate logistic regression analyses identified several risk factors for AKD, including older age, preexisting hypertension, congestive heart failure, cirrhosis, hepatorenal syndrome, recent diuretic use, elevated baseline eGFR, higher AKI stage, and dialysis-requiring AKI. To account for the competing risk of early mortality, Fine-Gray regression analyses further verified that AKD risk rises progressively with higher AKI stages. Survival analyses further demonstrated that, compared with patients achieving full recovery (stage 0 A/0B), the risk of *de novo* CKD increased progressively across all ADQI stages, with a significantly elevated risk observed even in stage 0 C.

AKD is operationally defined as persistent kidney dysfunction for 7–90 days [11]. In this study focusing specifically on the clinical continuum of AKD following AKI, 45.6% of patients with AKI progressed to AKD stages 1–3 rather than recovering, highlighting the poor prognosis associated with AKI and the substantial long-term healthcare burden for affected individuals. Previous studies in AKI populations have reported comparable AKD incidence rates. For example, a 5-year prospective study at Bordeaux University Hospital in France found that 47% of ICU patients with AKI developed AKD [21], while a study from Tayside & Fife, UK, including both community- and hospital-acquired AKI, reported an incidence of 33% [15]. Notably, these consistent epidemiological findings are limited to AKI populations. In broader populations, reported AKD incidence varies widely, influenced by factors such as the type of AKI (community- versus hospital-acquired), prior AKI history, and underlying comorbidities [22]. Despite such variability across studies, our data underscore a massive, often overlooked burden of persistent kidney impairment. Notably, 65.1% of patients not meeting conventional AKD stage 1–3 criteria were classified as ADQI stage 0 C [11]. This demonstrates the crucial clinical sensitivity of the ADQI framework in unmasking subclinical non-recovery that traditional binary definitions entirely overlook.

In this study, we explored factors influencing AKI progression to AKD. Hypertension has been associated with AKD, yet the clinical management of blood pressure during acute kidney injury remains highly complex. Existing evidence suggests that early antihypertensive treatment during the acute phase of AKI may worsen renal outcomes by reducing renal perfusion [23]. Similarly, intravenous antihypertensive therapy in patients with severe hypertension can exacerbate AKI [24]. Because our data captures baseline comorbidity rather than acute hemodynamic trajectories, preexisting hypertension should be interpreted cautiously as a marker of general cardiovascular vulnerability rather than a direct driver of AKD.

Hepatorenal syndrome emerged as a potent independent risk factor for AKD in our revised models. Hepatorenal syndrome represents a severe form of functional kidney impairment driven by progressive splanchnic vasodilation, which activates systemic vasoconstrictor pathways and leads to intense renal vasoconstriction [25]. Although historically considered a purely functional disorder without significant initial parenchymal injury, emerging evidence highlights that prolonged renal hypoperfusion and ongoing vasoconstriction can incite irreversible structural damage [26,27]. Consequently, delayed intervention for this sustained ischemic insult fundamentally impedes short-term recovery, driving the maladaptive transition toward acute kidney disease [27]. Particularly, dialysis-requiring AKI represents a severe clinical phenotype within the AKI-AKD-CKD continuum, where extensive nephron damage and acute loss of renal functional reserve naturally predispose patients to incomplete recovery and progression to AKD [28].

Data from this study also indicated that patients who received diuretics within 7 days prior to AKI onset were more likely to develop AKD. Although previous studies on the relationship between pre-AKI diuretic use and renal recovery are limited, existing evidence provides some insights. A systematic review of 64 studies concluded that the preventive role of diuretics in AKI is controversial. Although they may provide some protection against the need for kidney replacement therapy and mortality, their efficacy in treating established AKI is limited [29]. Furthermore, a multicenter survey in China identified diuretics as one of the most common drugs associated with hospital-acquired AKI [30]. While these studies focused on AKI, our findings extend this evidence to AKD. Rather than directly driving non-recovery, pre-AKI diuretic use likely serves as a clinical marker of underlying hemodynamic vulnerability and cardiorenal burden.

Interestingly, we observed a positive association between higher baseline eGFR and AKD development. A similar finding was reported in a cohort study conducted in Scotland, which included 56,906 participants [31]. This paradoxical association is strongly consistent with a detection bias interpretation: patients with higher baseline eGFR retain greater creatinine ‘headroom,’ meaning modest, persistent elevations more readily satisfy relative AKD criteria. Furthermore, rather than reflecting true biological risk, these findings may also be influenced by residual confounding and model structure.

Finally, the apparent protective effects of ICU admission, surgical procedures, and antibiotic use should be interpreted with caution, as they likely reflect residual confounding, surveillance bias, or model structure rather than true renoprotection. For instance, these observed associations may be attributed to the closer monitoring and more timely interventions these patients receive, which help prevent AKI from persisting. Furthermore, patients eligible for surgery typically have better baseline health and renal function reserves, making them more likely to recover from post-surgical AKI. Given confounding and competing risks, early risk stratification should rely on comprehensive clinical context over isolated parameters.

In our cohort, 19.8% of patients with AKD progressed to CKD within one year. Consistent with this trend, an Indian cohort of 200 children with AKD after AKI found that 16% had CKD stage ≥2 by 90 days [32]. We further show that, among patients without baseline CKD, AKD independently predicts *de novo* CKD, and this risk increases progressively across AKD severity, persisting even in patients with subclinical non-recovery (ADQI Stage 0 C). Supporting this, a cohort study of 17,049 veterans with baseline eGFR >60 mL/min/1.73 m^2^ reported that recovery time from AKI, regardless of its severity, was a strong predictor of stage 3 or higher CKD within one year [33]. Comparable findings were also observed in patients with postoperative AKI following cardiac surgery [34]. Unlike these studies that focused on AKI, our analysis specifically evaluates AKD, revealing its independent contribution to CKD progression and emphasizing the clinical importance of this continuum.

Our study has several notable strengths. While a substantial body of evidence already exists regarding the general epidemiology of AKD, this study leverages a remarkably large sample size derived from a decade of clinical data. This robust dataset provides the necessary statistical power to explore key determinants of AKD progression and explicitly validate the clinical importance of the harmonized ADQI staging framework. Specifically, our findings demonstrate the critical prognostic value of identifying subclinical non-recovery (Stage 0 C) in predicting *de novo* CKD progression, offering actionable insights for post-AKI risk stratification.

However, this study has several limitations. First, the study was performed at a single tertiary academic center in China. Because diagnostic and therapeutic practices vary by hospital and region, these findings may not generalize to AKI populations in other Chinese care settings. Secondly, as urine output data were not available, AKI was defined solely based on serum creatinine criteria according to KDIGO guidelines. This may lead to underestimation of AKI cases, as some patients with oliguria could have been missed. Thirdly, excluding patients without follow-up serum creatinine data introduced unavoidable selection and ascertainment bias. As detailed in Tables S1 and S4, the attrition patterns differed across assessment stages. For AKD assessment, patients with shorter hospital stays and fewer clinical interventions were disproportionately excluded due to a lack of serial creatinine measurements. This exclusion of milder acute cases likely overestimates the true incidence of AKD and inflates the apparent risk of non-recovery following AKI. Conversely, long-term CKD follow-up favored younger individuals, whereas older patients with severe acute comorbidities were frequently lost to follow-up, likely due to competing mortality risk or clinical severity. This loss of critically ill phenotypes inevitably biases long-term CKD surveillance and influences the estimated strength of the association between AKD and *de novo* CKD. Additionally, while the study sought to incorporate potentially relevant risk factors, the results may still be influenced by unmeasured confounding variables. Accordingly, multicenter prospective studies are warranted to validate these results, and additional work should explore strategies to improve renal outcomes after AKI.

## Conclusion

This retrospective study shows that nearly half of Chinese AKI survivors progress to AKD after 7 days, which is linked to a higher risk of *de novo* CKD, even among patients with subclinical non-recovery (Stage 0 C). These findings suggest that AKD following AKI may contribute to a measurable clinical burden, underscoring the potential value of post-discharge surveillance to manage such cases. Furthermore, our data highlight the importance of future efforts in risk-stratified tracking for patients with elevated clinical vulnerability.

## Supplementary Material

supplement_clean.docx

## Data Availability

The datasets generated and analyzed during the current study are not publicly available due to privacy and ethical restrictions. Further inquiries can be directed to the corresponding author.
