# Supplementary material for: Acute kidney disease after acute kidney injury: incidence, risk factors, and association with de novo chronic kidney disease
Source: Ren Fail. 2026 Oct 15;48(1):2745291. doi: 10.1080/0886022X.2026.2745291 (PMC13674642; doi:10.1080/0886022X.2026.2745291)
Supplement: Supplementary file 1 [file IRNF_A_2745291_SM6256.docx]

# Table S1. Baseline characteristics of AKD cohort patients included versus excluded for missing 7- to 90-day serum creatinine.

| Characteristics | Included Group | Excluded Group | P-value | SMD |
| --- | --- | --- | --- | --- |
| N | 23868 | 14172 |  |  |
| Age (median [IQR]) | 57.0 [47.0, 66.0] | 59.0 [50.0, 68.0] | <0.001 | 0.155 |
| Sex, male (%) | 15525 (65.0) | 9017 (63.6) | 0.005 | 0.03 |
| ICU admission (%) | 1921 (8.0) | 1673 (11.8) | <0.001 | 0.126 |
| Surgery (%) | 12542 (52.5) | 6017 (42.5) | <0.001 | 0.203 |
| Hypertension (%) | 8175 (34.3) | 4614 (32.6) | 0.001 | 0.036 |
| Diabetes (%) | 3753 (15.7) | 2224 (15.7) | 0.947 | 0.001 |
| Cancer (%) | 8744 (36.6) | 4851 (34.2) | <0.001 | 0.05 |
| Coronary artery disease (%) | 4872 (20.4) | 2318 (16.4) | <0.001 | 0.105 |
| Congestive heart failure (%) | 4692 (19.7) | 2396 (16.9) | <0.001 | 0.071 |
| Cirrhosis (%) | 1972 (8.3) | 1234 (8.7) | 0.136 | 0.016 |
| COPD (%) | 566 (2.4) | 425 (3.0) | <0.001 | 0.039 |
| Sepsis (%) | 1604 (6.7) | 1137 (8.0) | <0.001 | 0.05 |
| Hypovolemia (%) | 424 (1.8) | 303 (2.1) | 0.014 | 0.026 |
| Obstruction (%) | 363 (1.5) | 268 (1.9) | 0.007 | 0.029 |
| Cardiorenal syndrome (%) | 1056 (4.4) | 708 (5.0) | 0.011 | 0.027 |
| Hepatorenal syndrome (%) | 541 (2.3) | 649 (4.6) | <0.001 | 0.127 |
| Nephrotoxins (%) | 16073 (67.3) | 8983 (63.4) | <0.001 | 0.083 |
| ACEI/ARB (%) | 2742 (11.5) | 1431 (10.1) | <0.001 | 0.045 |
| Diuretic (%) | 15040 (63.0) | 7440 (52.5) | <0.001 | 0.214 |
| Metformin (%) | 398 (1.7) | 247 (1.7) | 0.61 | 0.006 |
| Statin (%) | 3591 (15.0) | 1918 (13.5) | <0.001 | 0.043 |
| Antibiotics (%) | 19873 (83.3) | 11619 (82.0) | 0.002 | 0.034 |
| Baseline eGFR, mL/min/1.73 m2 (median [IQR]) | 100.3 [68.6, 116.1] | 96.5 [63.7, 111.7] | <0.001 | 0.113 |
| Baseline eGFR group, mL/min/1.73 m2 (%) |  |  | <0.001 | 0.091 |
| <30 | 1720 (7.2) | 1072 (7.6) |  |  |
| 30–44 | 1500 (6.3) | 1078 (7.6) |  |  |
| 45–59 | 1666 (7.0) | 1113 (7.9) |  |  |
| 60–89 | 4217 (17.7) | 2746 (19.4) |  |  |
| ≥90 | 14765 (61.9) | 8163 (57.6) |  |  |
| Peak AKI stage within 7 days post AKI (%) |  |  | <0.001 | 0.123 |
| Stage 1 | 15831 (66.3) | 10132 (71.5) |  |  |
| Stage 2 | 4519 (18.9) | 2458 (17.3) |  |  |
| Stage 3 | 3518 (14.7) | 1582 (11.2) |  |  |
| Dialysis-requiring AKI (%) | 606 (2.5) | 178 (1.3) | <0.001 | 0.094 |
| Hospital length of stay (median [IQR]) | 21.0 [14.0, 31.0] | 9.0 [5.0, 15.0] | <0.001 | 0.952 |

Data are presented as median [interquartile range (IQR)] for continuous variables or number (percentage) for categorical variables.

Abbreviations: SMD: Standardized Mean Difference; ICU: Intensive Care Unit; ACEI: Angiotensin-converting enzyme inhibitors; ARB: Angiotensin II receptor blockers; COPD: Chronic Obstructive Pulmonary Disease; eGFR: Estimated glomerular filtration rate; SCr: Serum creatinine.

# Table S2. Baseline characteristics of participants categorized by AKD stage

| Characteristics | Overall | AKD Stage 0A/0B | AKD Stage 0C | AKD Stage 1 | AKD Stage 2 | AKD Stage 3 | P-value | Average pairwise SMD |
| --- | --- | --- | --- | --- | --- | --- | --- | --- |
| N | 23868 | 4528 | 8456 | 4420 | 3000 | 3464 |  |  |
| Age (median [IQR]) | 57.0 [47.0, 66.0] | 58.0 [47.0, 67.0] | 57.0 [48.0, 66.0] | 56.0 [46.0, 65.0] | 56.0 [47.0, 66.0] | 56.0 [47.0, 65.0] | <0.001 | 0.054 |
| Sex, male (%) | 15525 (65.0) | 3060 (67.6) | 5581 (66.0) | 2752 (62.3) | 1902 (63.4) | 2230 (64.4) | <0.001 | 0.056 |
| ICU admission (%) | 1921 (8.0) | 671 (14.8) | 579 (6.8) | 278 (6.3) | 188 (6.3) | 205 (5.9) | <0.001 | 0.123 |
| Surgery (%) | 12542 (52.5) | 2024 (44.7) | 4677 (55.3) | 2284 (51.7) | 1418 (47.3) | 2139 (61.7) | <0.001 | 0.17 |
| Hypertension (%) | 8175 (34.3) | 1563 (34.5) | 2975 (35.2) | 1458 (33.0) | 1025 (34.2) | 1154 (33.3) | 0.095 | 0.024 |
| Diabetes (%) | 3753 (15.7) | 753 (16.6) | 1364 (16.1) | 683 (15.5) | 490 (16.3) | 463 (13.4) | 0.001 | 0.041 |
| Cancer (%) | 8744 (36.6) | 1408 (31.1) | 3419 (40.4) | 1769 (40.0) | 1076 (35.9) | 1072 (30.9) | <0.001 | 0.117 |
| Coronary artery disease (%) | 4872 (20.4) | 849 (18.8) | 1685 (19.9) | 832 (18.8) | 605 (20.2) | 901 (26.0) | <0.001 | 0.077 |
| Congestive heart failure (%) | 4692 (19.7) | 815 (18.0) | 1387 (16.4) | 817 (18.5) | 664 (22.1) | 1009 (29.1) | <0.001 | 0.143 |
| Cirrhosis (%) | 1972 (8.3) | 266 (5.9) | 729 (8.6) | 398 (9.0) | 316 (10.5) | 263 (7.6) | <0.001 | 0.079 |
| COPD (%) | 566 (2.4) | 122 (2.7) | 182 (2.2) | 90 (2.0) | 78 (2.6) | 94 (2.7) | 0.079 | 0.025 |
| Sepsis (%) | 1604 (6.7) | 513 (11.3) | 387 (4.6) | 233 (5.3) | 207 (6.9) | 264 (7.6) | <0.001 | 0.121 |
| Hypovolemia (%) | 424 (1.8) | 137 (3.0) | 113 (1.3) | 59 (1.3) | 54 (1.8) | 61 (1.8) | <0.001 | 0.054 |
| Obstruction (%) | 363 (1.5) | 116 (2.6) | 119 (1.4) | 48 (1.1) | 46 (1.5) | 34 (1.0) | <0.001 | 0.056 |
| Cardiorenal syndrome (%) | 1056 (4.4) | 230 (5.1) | 375 (4.4) | 203 (4.6) | 130 (4.3) | 118 (3.4) | 0.009 | 0.036 |
| Hepatorenal syndrome (%) | 541 (2.3) | 76 (1.7) | 132 (1.6) | 110 (2.5) | 92 (3.1) | 131 (3.8) | <0.001 | 0.074 |
| Nephrotoxins (%) | 16073 (67.3) | 2801 (61.9) | 5754 (68.0) | 3038 (68.7) | 2014 (67.1) | 2466 (71.2) | <0.001 | 0.086 |
| ACEI/ARB (%) | 2742 (11.5) | 445 (9.8) | 1037 (12.3) | 522 (11.8) | 328 (10.9) | 410 (11.8) | 0.001 | 0.037 |
| Diuretic (%) | 15040 (63.0) | 2686 (59.3) | 5117 (60.5) | 2685 (60.7) | 1984 (66.1) | 2568 (74.1) | <0.001 | 0.15 |
| Metformin (%) | 398 (1.7) | 74 (1.6) | 149 (1.8) | 70 (1.6) | 51 (1.7) | 54 (1.6) | 0.917 | 0.008 |
| Statin (%) | 3591 (15.0) | 603 (13.3) | 1349 (16.0) | 621 (14.0) | 422 (14.1) | 596 (17.2) | <0.001 | 0.054 |
| Antibiotics (%) | 19873 (83.3) | 3903 (86.2) | 6959 (82.3) | 3592 (81.3) | 2468 (82.3) | 2951 (85.2) | <0.001 | 0.069 |
| Baseline eGFR, mL/min/1.73 m2 (median [IQR]) | 100.3 [68.6, 116.1] | 71.5 [40.7, 98.4] | 100.0 [75.0, 112.0] | 105.8 [80.3, 118.8] | 106.9 [74.2, 123.3] | 116.0 [91.7, 141.5] | <0.001 | 0.545 |
| Baseline eGFR group, mL/min/1.73 m2 (%) |  |  |  |  |  |  | <0.001 | 0.442 |
| <30 | 1720 (7.2) | 726 (16.0) | 467 (5.5) | 265 (6.0) | 206 (6.9) | 56 (1.6) |  |  |
| 30–44 | 1500 (6.3) | 556 (12.3) | 397 (4.7) | 227 (5.1) | 206 (6.9) | 114 (3.3) |  |  |
| 45–59 | 1666 (7.0) | 538 (11.9) | 541 (6.4) | 225 (5.1) | 181 (6.0) | 181 (5.2) |  |  |
| 60–89 | 4217 (17.7) | 1145 (25.3) | 1598 (18.9) | 601 (13.6) | 391 (13.0) | 482 (13.9) |  |  |
| ≥90 | 14765 (61.9) | 1563 (34.5) | 5453 (64.5) | 3102 (70.2) | 2016 (67.2) | 2631 (76.0) |  |  |
| Peak AKI stage within 7 days post AKI (%) |  |  |  |  |  |  | <0.001 | 1.065 |
| Stage 1 | 15831 (66.3) | 3940 (87.0) | 7022 (83.0) | 3094 (70.0) | 1208 (40.3) | 567 (16.4) |  |  |
| Stage 2 | 4519 (18.9) | 477 (10.5) | 1156 (13.7) | 1066 (24.1) | 1219 (40.6) | 601 (17.3) |  |  |
| Stage 3 | 3518 (14.7) | 111 (2.5) | 278 (3.3) | 260 (5.9) | 573 (19.1) | 2296 (66.3) |  |  |
| Dialysis-requiring AKI (%) | 606 (2.5) | 71 (1.6) | 75 (0.9) | 88 (2.0) | 90 (3.0) | 282 (8.1) | <0.001 | 0.167 |
| Hospital length of stay (median [IQR]) | 21.0 [14.0, 31.0] | 19.0 [12.0, 29.0] | 21.0 [13.0, 30.0] | 21.0 [13.0, 30.0] | 23.0 [15.0, 34.0] | 25.5 [18.0, 35.0] | <0.001 | 0.167 |

Data are presented as median [interquartile range (IQR)] for continuous variables or number (percentage) for categorical variables.

Average pairwise SMD represents the mean of standardized mean differences across all pairwise comparisons among AKD stage groups.

Abbreviations: SMD: Standardized Mean Difference; ICU: Intensive Care Unit; ACEI: Angiotensin-converting enzyme inhibitors; ARB: Angiotensin II receptor blockers; COPD: Chronic Obstructive Pulmonary Disease; eGFR: Estimated glomerular filtration rate; SCr: Serum creatinine.

# Table S3. Association between AKI stage and subsequent AKD using Fine-Gray competing-risk regression.

| **Characteristics** | **Crude model^a^** | | | **Adjusted model^b^** | | |
| --- | --- | --- | --- | --- | --- | --- |
|  | **sHR** | **95%CI** | **P-value** | **sHR** | **95%CI** | **P-value** |
| AKI Stages(versus Stage 1) |  |  |  |  |  |  |
| Stage 2 | 2.54 | 2.43, 2.66 | <0.001 | 2.33 | 2.23, 2.45 | <0.001 |
| Stage 3 | 4.73 | 4.52, 4.95 | <0.001 | 3.54 | 3.36, 3.72 | <0.001 |

a The Crude model is unadjusted.

b The Adjusted model controls for Age, Sex, ICU admission, Surgery, Hypertension, Diabetes, Cancer, Coronary artery disease, Congestive heart failure, Cirrhosis, Hypovolemia, Obstruction, Cardiorenal syndrome, Hepatorenal syndrome, Nephrotoxins, Diuretic, Antibiotics, Baseline eGFR, Dialysis-requiring AKI.

Abbreviations: sHR, subdistribution hazard ratio; CI, confidence interval.

# Table S4. Baseline characteristics of CKD cohort patients included versus excluded for missing 90- to 365-day serum creatinine.

| Characteristics | Included Group | Excluded Group | P-value | SMD |
| --- | --- | --- | --- | --- |
| N | 6597 | 11592 |  |  |
| Age (median [IQR]) | 53.0 [43.0, 61.0] | 57.0 [48.0, 65.0] | <0.001 | 0.303 |
| Sex, male (%) | 4277 (64.8) | 7538 (65.0) | 0.803 | 0.004 |
| ICU admission (%) | 223 (3.4) | 873 (7.5) | <0.001 | 0.184 |
| Surgery (%) | 3940 (59.7) | 6864 (59.2) | 0.51 | 0.01 |
| Hypertension (%) | 1833 (27.8) | 3343 (28.8) | 0.134 | 0.023 |
| Diabetes (%) | 792 (12.0) | 1462 (12.6) | 0.242 | 0.018 |
| Cancer (%) | 3211 (48.7) | 3863 (33.3) | <0.001 | 0.316 |
| Coronary artery disease (%) | 1006 (15.2) | 2292 (19.8) | <0.001 | 0.119 |
| Congestive heart failure (%) | 871 (13.2) | 2086 (18.0) | <0.001 | 0.132 |
| Cirrhosis (%) | 737 (11.2) | 929 (8.0) | <0.001 | 0.107 |
| COPD (%) | 89 (1.3) | 226 (1.9) | 0.003 | 0.047 |
| Sepsis (%) | 203 (3.1) | 737 (6.4) | <0.001 | 0.155 |
| Hypovolemia (%) | 66 (1.0) | 235 (2.0) | <0.001 | 0.084 |
| Obstruction (%) | 61 (0.9) | 112 (1.0) | 0.843 | 0.004 |
| Cardiorenal syndrome (%) | 160 (2.4) | 333 (2.9) | 0.082 | 0.028 |
| Hepatorenal syndrome (%) | 78 (1.2) | 325 (2.8) | <0.001 | 0.116 |
| Nephrotoxins (%) | 4973 (75.4) | 7709 (66.5) | <0.001 | 0.197 |
| ACEI/ARB (%) | 693 (10.5) | 1146 (9.9) | 0.192 | 0.02 |
| Diuretic (%) | 3871 (58.7) | 7303 (63.0) | <0.001 | 0.089 |
| Metformin (%) | 100 (1.5) | 185 (1.6) | 0.722 | 0.006 |
| Statin (%) | 817 (12.4) | 1554 (13.4) | 0.052 | 0.03 |
| Antibiotics (%) | 5382 (81.6) | 10065 (86.8) | <0.001 | 0.144 |
| Baseline eGFR, mL/min/1.73 m2 (median [IQR]) | 109.1 [96.1, 121.7] | 106.0 [92.2, 119.5] | <0.001 | 0.106 |
| Baseline eGFR group, mL/min/1.73 m2 (%) |  |  | <0.001 | 0.108 |
| 60–89 | 1211 (18.4) | 2631 (22.7) |  |  |
| ≥90 | 5386 (81.6) | 8961 (77.3) |  |  |
| Peak AKI stage within 7 days post AKI (%) |  |  | <0.001 | 0.135 |
| Stage 1 | 4434 (67.2) | 7096 (61.2) |  |  |
| Stage 2 | 1214 (18.4) | 2333 (20.1) |  |  |
| Stage 3 | 949 (14.4) | 2163 (18.7) |  |  |
| Dialysis-requiring AKI (%) | 77 (1.2) | 215 (1.9) | <0.001 | 0.056 |
| Hospital length of stay (median [IQR]) | 23.0 [14.0, 34.0] | 23.0 [15.0, 32.0] | 0.763 | 0.091 |
| AKD stage (%) |  |  | <0.001 | 0.229 |
| Stage 0A/0B | 739 (11.2) | 1853 (16.0) |  |  |
| Stage 0C | 2684 (40.7) | 4090 (35.3) |  |  |
| Stage 1 | 1479 (22.4) | 2076 (17.9) |  |  |
| Stage 2 | 832 (12.6) | 1452 (12.5) |  |  |
| Stage 3 | 863 (13.1) | 2121 (18.3) |  |  |

Data are presented as median [interquartile range (IQR)] for continuous variables or number (percentage) for categorical variables.

Abbreviations: SMD: Standardized Mean Difference; ICU: Intensive Care Unit; ACEI: Angiotensin-converting enzyme inhibitors; ARB: Angiotensin II receptor blockers; COPD: Chronic Obstructive Pulmonary Disease; eGFR: Estimated glomerular filtration rate; SCr: Serum creatinine.

# Table S5. Baseline characteristics of participants categorized by CKD status

| Characteristics | CKD | No CKD | P-value | SMD |
| --- | --- | --- | --- | --- |
| N | 1308 | 5289 |  |  |
| Age (median [IQR]) | 55.0 [46.0, 64.0] | 53.0 [43.0, 60.0] | <0.001 | 0.248 |
| Sex, male (%) | 829 (63.4) | 3448 (65.2) | 0.231 | 0.038 |
| ICU admission (%) | 43 (3.3) | 180 (3.4) | 0.903 | 0.006 |
| Surgery (%) | 692 (52.9) | 3248 (61.4) | <0.001 | 0.173 |
| Hypertension (%) | 479 (36.6) | 1354 (25.6) | <0.001 | 0.24 |
| Diabetes (%) | 234 (17.9) | 558 (10.6) | <0.001 | 0.211 |
| Cancer (%) | 631 (48.2) | 2580 (48.8) | 0.75 | 0.011 |
| Coronary artery disease (%) | 231 (17.7) | 775 (14.7) | 0.008 | 0.082 |
| Congestive heart failure (%) | 201 (15.4) | 670 (12.7) | 0.011 | 0.078 |
| Cirrhosis (%) | 149 (11.4) | 588 (11.1) | 0.816 | 0.009 |
| COPD (%) | 27 (2.1) | 62 (1.2) | 0.018 | 0.071 |
| Sepsis (%) | 56 (4.3) | 147 (2.8) | 0.006 | 0.081 |
| Hypovolemia (%) | 14 (1.1) | 52 (1.0) | 0.898 | 0.009 |
| Obstruction (%) | 15 (1.1) | 46 (0.9) | 0.438 | 0.028 |
| Cardiorenal syndrome (%) | 50 (3.8) | 110 (2.1) | <0.001 | 0.103 |
| Hepatorenal syndrome (%) | 16 (1.2) | 62 (1.2) | 0.992 | 0.005 |
| Nephrotoxins (%) | 960 (73.4) | 4013 (75.9) | 0.067 | 0.057 |
| ACEI/ARB (%) | 202 (15.4) | 491 (9.3) | <0.001 | 0.188 |
| Diuretic (%) | 816 (62.4) | 3055 (57.8) | 0.003 | 0.095 |
| Metformin (%) | 32 (2.4) | 68 (1.3) | 0.003 | 0.086 |
| Statin (%) | 209 (16.0) | 608 (11.5) | <0.001 | 0.131 |
| Antibiotics (%) | 1053 (80.5) | 4329 (81.8) | 0.279 | 0.034 |
| Baseline eGFR, mL/min/1.73 m2 (median [IQR]) | 96.1 [78.3, 110.7] | 111.2 [99.9, 123.9] | <0.001 | 0.732 |
| Baseline eGFR group, mL/min/1.73 m2 (%) |  |  | <0.001 | 0.651 |
| 60–89 | 527 (40.3) | 684 (12.9) |  |  |
| ≥90 | 781 (59.7) | 4605 (87.1) |  |  |
| Peak AKI stage within 7 days post AKI (%) |  |  | 0.002 | 0.107 |
| Stage 1 | 829 (63.4) | 3605 (68.2) |  |  |
| Stage 2 | 258 (19.7) | 956 (18.1) |  |  |
| Stage 3 | 221 (16.9) | 728 (13.8) |  |  |
| Dialysis-requiring AKI (%) | 38 (2.9) | 39 (0.7) | <0.001 | 0.163 |
| Hospital length of stay (median [IQR]) | 21.0 [13.0, 33.0] | 23.0 [15.0, 34.0] | 0.003 | 0.033 |
| AKD stage (%) |  |  | <0.001 | 0.257 |
| Stage 0A/0B | 110 (8.4) | 629 (11.9) |  |  |
| Stage 0C | 451 (34.5) | 2233 (42.2) |  |  |
| Stage 1 | 313 (23.9) | 1166 (22.0) |  |  |
| Stage 2 | 191 (14.6) | 641 (12.1) |  |  |
| Stage 3 | 243 (18.6) | 620 (11.7) |  |  |

Data are presented as median [interquartile range (IQR)] for continuous variables or number (percentage) for categorical variables.

Abbreviations: SMD: Standardized Mean Difference; ICU: Intensive Care Unit; ACEI: Angiotensin-converting enzyme inhibitors; ARB: Angiotensin II receptor blockers; COPD: Chronic Obstructive Pulmonary Disease; eGFR: Estimated glomerular filtration rate; SCr: Serum creatinine.

# Table S6. Univariate Cox regression analysis of risk factors for *de novo* CKD

| **Risk factors** | **Crude model^a^** | |
| --- | --- | --- |
|  | **HR (95% CI)** | **P -value** |
| AKD | 1.51 (1.36-1.69) | <.001 |
| AKD Stages(versus Stage 0A/0B) |  |  |
| Stage 0C | 1.14 (0.92-1.40) | 0.232 |
| Stage 1 | 1.48 (1.19-1.83) | <.001 |
| Stage 2 | 1.62 (1.28-2.05) | <.001 |
| Stage 3 | 2.08 (1.66-2.61) | <.001 |
| Age | 1.02 (1.01-1.02) | <.001 |
| Sex, male | 0.93 (0.84-1.05) | 0.241 |
| ICU admission | 0.98 (0.72-1.32) | 0.87 |
| Surgery | 0.73 (0.65-0.81) | <.001 |
| Hypertension | 1.58 (1.41-1.77) | <.001 |
| Diabetes | 1.68 (1.46-1.94) | <.001 |
| Cancer | 0.98 (0.88-1.09) | 0.699 |
| Coronary artery disease | 1.21 (1.05-1.39) | 0.01 |
| Congestive heart failure | 1.22 (1.05-1.42) | 0.01 |
| Cirrhosis | 1.02 (0.86-1.21) | 0.833 |
| COPD | 1.59 (1.09-2.33) | 0.017 |
| Sepsis | 1.49 (1.14-1.95) | 0.003 |
| Hypovolemia | 1.05 (0.62-1.79) | 0.844 |
| Obstruction | 1.28 (0.77-2.12) | 0.346 |
| Cardiorenal syndrome | 1.72 (1.30-2.29) | <.001 |
| Hepatorenal syndrome | 1.07 (0.65-1.75) | 0.797 |
| Nephrotoxins | 0.89 (0.78-1.00) | 0.055 |
| ACEI/ARB | 1.66 (1.43-1.93) | <.001 |
| Diuretic | 1.19 (1.06-1.33) | 0.002 |
| Metformin | 1.71 (1.20-2.42) | 0.003 |
| Statin | 1.39 (1.20-1.61) | <.001 |
| Antibiotics | 0.93 (0.81-1.06) | 0.276 |
| Baseline eGFR, mL/min/1.73 m2 | 0.97 (0.97-0.97) | <.001 |
| Baseline eGFR group, mL/min/1.73 m2(versus 60–89) |  |  |
| ≥90 | 0.27 (0.24-0.31) | <.001 |
| Peak AKI stage within 7 days post AKI(versus Stage 1) |  |  |
| Stage 2 | 1.16 (1.01-1.33) | 0.042 |
| Stage 3 | 1.29 (1.11-1.50) | <.001 |
| Dialysis-requiring AKI | 3.11 (2.25-4.30) | <.001 |

a The Crude model is unadjusted.

Abbreviations: ICU: Intensive Care Unit; ACEI: Angiotensin-converting enzyme inhibitors; ARB: Angiotensin II receptor blockers; COPD: Chronic Obstructive Pulmonary Disease; eGFR: Estimated glomerular filtration rate; SCr: Serum creatinine.
